# Effect of Co-inhabiting Coagulase Negative Staphylococci on *S. aureus agr* Quorum Sensing, Host Factor Binding, and Biofilm Formation

**DOI:** 10.3389/fmicb.2019.02212

**Published:** 2019-09-27

**Authors:** Pai Peng, Mara Baldry, Bengt H. Gless, Martin S. Bojer, Carmen Espinosa-Gongora, Sharmin J. Baig, Paal S. Andersen, Christian A. Olsen, Hanne Ingmer

**Affiliations:** ^1^Faculty of Health and Medical Sciences, Department of Veterinary and Animal Sciences, University of Copenhagen, Copenhagen, Denmark; ^2^Faculty of Health and Medical Sciences, Department of Drug Design and Pharmacology, University of Copenhagen, Copenhagen, Denmark; ^3^Department of Bacteria, Parasites and Fungi, Statens Serum Institut, Copenhagen, Denmark

**Keywords:** *Staphylococcus aureus*, coagulase-negative staphylococci, colonization, *agr*, quorum sensing interaction, cross-talk

## Abstract

*Staphylococcus aureus* is a commensal colonizer of both humans and animals, but also an opportunistic pathogen responsible for a multitude of diseases. In recent years, colonization of pigs by methicillin resistant *S. aureus* has become a problem with increasing numbers of humans being infected by livestock strains. In *S. aureus* colonization and virulence factor expression is controlled by the *agr* quorum sensing system, which responds to and is activated by self-generated, autoinducing peptides (AIPs). AIPs are also produced by coagulase negative staphylococci (CoNS) commonly found as commensals in both humans and animals, and interestingly, some of these inhibit *S. aureus agr* activity. Here, we have addressed if cross-communication occurs between *S. aureus* and CoNS strains isolated from pig nares, and if so, how properties such as host factor binding and biofilm formation are affected. From 25 pig nasal swabs we obtained 54 staphylococcal CoNS isolates belonging to 8 different species. Of these, none were able to induce *S. aureus agr* as monitored by reporter gene fusions to *agr* regulated genes but a number of *agr*-inhibiting species were identified including *Staphylococcus hyicus*, *Staphylococcus simulans*, *Staphylococcus arlettae*, *Staphylococcus lentus*, and *Staphylococcus chromogenes*. After establishing that the inhibitory activity was mediated via AgrC, the receptor of AIPs, we synthesized selective AIPs to explore their effect on adhesion of *S. aureus* to fibronectin, a host factor involved in *S. aureus* colonization. Here, we found that the CoNS AIPs did not affect adhesion of *S. aureus* except for strain 8325-4. When individual CoNS strains were co-cultured together with *S. aureus* we observed variable degrees of biofilm formation which did not correlate with *agr* interactions. Our results show that multiple CoNS species can be isolated from pig nares and that the majority of these produce AIPs that inhibit *S. aureus agr*. Further they show that the consequences of the interactions between CoNS and *S. aureus* are complex and highly strain dependent.

## Introduction

*Staphylococcus aureus* is a common colonizer and opportunistic pathogen of both animals and humans. The increasing spread of antibiotic resistance among *S. aureus* strains is of major concern in the treatment of staphylococcal infections, with methicillin-resistant *S. aureus* (MRSA) in particular being a proven health risk to humans, causing skin and soft tissue infections, food poisoning, and even fatal systemic disease (Fridkin et al., 2005; Kourbatova et al., 2005; King et al., 2006). MRSA strains are commonly divided into community, hospital or livestock associated and in recent years, the transmission of livestock-associated (LA)-MRSA from animals to humans has become a public health concern particularly in Europe, North America and Asia where pig farming is extensive. Within the EU alone nearly 46% of pigs are colonized by strains of the most predominant LA-MRSA type namely the clonal complex 398 (CC398) (Khanna et al., 2008; Lewis et al., 2008; Van Duijkeren et al., 2008; Authority, 2009; Smith et al., 2009; Golding et al., 2010; Köck et al., 2013; Chuang and Huang, 2015). Studies have revealed a high prevalence of nasal MRSA carriage in pig slaughterhouse workers and pig farmers, indicating that working with MRSA-colonized pigs is the predominant risk factor (Lewis et al., 2008; Van Cleef et al., 2010).

In general, *S. aureus* colonization is a multifactorial process involving a number of adhesins or host binding proteins that are expressed by, and located on, the surface of the bacterium (Josse et al., 2017). Particularly fibronectin binding proteins have been reported to be important for internalization and uptake of *S. aureus* by keratinocytes; to be key in the adhesion of *S. aureus* to keratinocytes of atopic skin and also to contribute to biofilm formation by MRSA strains (Cho et al., 2001; Kintarak et al., 2004; O’Neill et al., 2008; Josse et al., 2017). In addition to colonization factors, *S. aureus* also expresses a multitude of toxins and other factors necessary for virulence and biofilm formation (Archer et al., 2011; Kobayashi et al., 2015). Production of both adhesins and toxins are controlled by the accessory gene regulator (*agr*) quorum sensing system with the former being produced at low bacterial cell densities and the latter at high cell densities (Yarwood and Schlievert, 2003). *agr* is composed of a two component system which senses a self-generated autoinducing peptide (AIP) that, by binding to the sensor histidine kinase AgrC, leads to phosphorylation of the AgrA response-regulator and expression of the main *agr* effector molecule, RNAIII. As cells enter stationary phase, RNAIII is responsible for the down-regulation of host binding proteins such as Protein A encoded by *spa* and the concomitant upregulation of toxins such as α-hemolysin encoded by *hla* (Queck et al., 2008; Wang et al., 2014; Le and Otto, 2015). An RNAIII-independent *agr* gene regulation pathway also exists, involving AgrA-mediated expression of a family of toxins called the phenol soluble modulins (PSMs) (Periasamy et al., 2012). These PSMs are important players in biofilm formation and dispersal linking *agr* and biofilm formation (Boles and Horswill, 2008; Periasamy et al., 2012). Interestingly *agr* varies between *S. aureus* strains and can be divided into four groups (AgrC-I-IV) where AIPs from the corresponding group lead to self-activation whereas AIPs from other groups lead to cross-inhibition (Otto et al., 2001; Olson et al., 2014; Le and Otto, 2015). This group specificity has lead to an interest in studying the inhibitory activity of non-cognate AIPs as antivirulence sources targeting *agr* (Canovas et al., 2016; Tal-Gan et al., 2016).

Humans and animals are also colonized with a variety of other staphylococcal species. In contrast to *S. aureus* they do not produce coagulase and thus are termed the coagulase negative staphylococci (CoNS). Commonly, the CoNS are not pathogens and their presence has been suggested to influence *S. aureus* colonization. For example, in humans it has been proposed that *Staphylococcus epidermidis* may prevent colonization by *S. aureus* (Iwase et al., 2010), whereas in pigs, *S. aureus* colonization was not observed in the presence of *Staphylococcus sciuri*, *Staphylococcus cohnii*, or *Staphylococcus saprophyticus* (Verstappen et al., 2017). Interestingly, CoNS also encode AIP-like molecules and some of these are able to inhibit *S. aureus agr* (Otto et al., 1999; Canovas et al., 2016; Gless et al., 2017, 2019; Mahmmod et al., 2018). This cross-talk has been suggested to be involved in the competition between *S. aureus* and *S. epidermidis* on the skin (Otto et al., 2001) and in preventing MRSA colonization (Paharik et al., 2017).

The *agr*-mediated interactions between species isolated from the same host niche remains largely unexplored. In the present study we have examined which staphylococcal species co-colonize the pig nares and assessed the extent to which isolated CoNS strains are able to inhibit *S. aureus agr*. We have addressed if *agr* mediated cross-species communication affects *S. aureus* binding to fibronectin as well as biofilm formation, both elements that may be important for host colonization. Our results suggest extensive cross-communication between CoNS and *S. aureus* colonizing the same host niche. A better understanding of the role of *agr* cross-talk between colonizing staphylococci may provide insightful information that can be used for future exploitation in *S. aureus* colonization interference and anti-virulence therapy.

## Materials and Methods

### Bacterial Strains and Growth Conditions

Strains used in this study are listed in Table 1. Unless otherwise stated, all bacterial strains were grown in Tryptone Soya Broth (TSB) from Oxoid, at a 1:10 volume/flask ratio, at 37°C with shaking at 200 rpm.

**TABLE 1:** Strains in this study and their source.

**Strain**	**Description**	**References**
8325-4	*S. aureus* producing AIP group I	Novick and Morse, 1967
PC203	*S. aureus* 8325-4, *spa:lacZ*	Chan and Foster, 1998
PC322	*S. aureus* 8325-4, *hla:lacZ*	Chan and Foster, 1998
SH101F7	*S. aureus* 8325-4, *rnaIII:lacZ*	Horsburgh et al., 2002
RN10829/p*agrC*-I	*S. aureus* AgrC-I P3*:blaZ*	Nielsen et al., 2014
RN10829/p*agrC*-II	*S. aureus* AgrC-II P3*:blaZ*	Gless et al., 2019
RN10829/p*agrC*-III	*S. aureus* AgrC-III P3*:blaZ*	Gless et al., 2019
RN10829/p*agrC*-IV	*S. aureus* AgrC-IV P3*:blaZ*	Gless et al., 2019
RN10829/p*agrC*-I-R23H (AgrC const.)	*S. aureus* AgrC-I-R23H P3*:blaZ*	Geisinger et al., 2009
RN6607	*S. aureus* producing AIP group II	(Lab stock)
MW2	*S. aureus* producing AIP group III	(Lab stock)
RN4850	*S. aureus* producing AIP group IV	(Lab stock)
61599	LA-MRSA CC398 strain	Tang et al., 2017b
8325-4Δ*agr*	Transduction from *S. aureus* RN6911	Canovas et al., 2016
HG001	*S. aureus* 8325-4, restored *rsbU*	Herbert et al., 2010
HG003	*S. aureus* 8325-4, restored *rsbU* and *tcaR*	Herbert et al., 2010

### Sample Collection, Isolation, and Identification

Nasal swabs (E-Swab, Copan Diagnostics Inc., United States) were collected from the pig nasal cavity of randomly selected pigs (weighing 20–30 kg) at three organic farms in Denmark. It should be noted that no permission is required to sample the nostril of pigs according to the Danish Animal Experimentation Act § 1.2. Samples were sent within 24 h to the Department of Veterinary and Animal Sciences, University of Copenhagen, and analyzed on the day of arrival. In total, 25 samples from 25 pigs were analyzed. Swabs were suspended and diluted in saline solution and plated on SaSelect^TM^ plates (Bio-Rad) for staphylococcal species isolation. Species identification was carried out by matrix-assisted laser desorption ionization–time of flight mass spectrometry (MALDI-TOF MS) and *tuf* gene analysis by standard PCR-based methods (Hwang et al., 2011).

### β-Galactosidase Plate Assay

This assay was performed as previously described (Nielsen et al., 2010; Bojer et al., 2017). Briefly, the fused reporter strains PC203 (*spa*:*lacZ*), PC322 (*hla*:*lacZ*), and SH101F7 (*rnaIII*:*lacZ*) all in the 8325-4 strain background, were grown in TSA agar supplemented with 150 μg/mL β-galactosidase substrate 5-bromo-4-chloro-3-indolyl-b-D-galactopyranoside (X-gal) and 5 μg/mL erythromycin. Sixty microliter supernatants of the identified staphylococcal strains or TSB medium were added to premade wells in the plates. The plates were incubated at 37°C for 10–24 h (the incubation time varies depending on the different reporter strains) until the plates appeared blue.

### β-Lactamase Assay

This method was carried out as previously described with minor modifications (Nielsen et al., 2014; Bojer et al., 2017). Briefly, the reporter strains RN10829/p*agrC*-I-IV (WT) and RN10829/p*agrC-*I-R23H (AgrC const.) were treated with a 1/10 volume of CoNS supernatant at OD600 = 0.35, followed by the addition of a 1/10 volume of AIP-I-IV containing supernatant obtained separately from *S. aureus* strain 8325-4, RN6607, MW2 and RN4850. For the experiment investigating whether CoNS supernatants can induce *agr*, external AIP-I-IV supernatants were added as activation controls only. Samples were obtained after incubating at 37°C for 1 h, and optical density at 600 nm was recorded. Samples were stored at −80°C before thawing to test for β-lactamase activity as described (Bojer et al., 2017). Activity was calculated as arbitrary units based on nitrocefin conversion velocities (*V*_*max*_, ΔOD486 nm/time) normalized to the sample cell densities.

### Chemical Synthesis of AIPs

All AIPs were synthesized according to a previously reported protocol (Gless et al., 2017). Briefly, linear peptides were synthesized using automated 9-fluorenylmethyloxycarbonyl (Fmoc) solid-phase peptide synthesis (SPPS) on a Gly-ChemMatrix resin loaded with Fmoc-3-amino-4-(methylamino)-benzoic acid (Fmoc-MeDbz-OH). The last residue was incorporated as *N*-Boc protected amino acid. After SPPS, the MeDbz linker was converted to the *N*-acyl-benzimidazolinone (Nbz) species by treating the resin with 4-nitrophenyl-chloroformate in dichloromethane followed by a solution of *i*-Pr_2_NEt in dimethylformamide. The activated Nbz-resin was then treated with a trifluoroacetic acid (TFA) solution to cleave protecting groups and after excessive washing, swelled in cyclization buffer (phosphate buffer, 0.2 M, pH 6.8 in 50% acetonitrile) and incubated at 50°C for 2 h. The AIP containing solution was separated from the resin and the desired AIP purified by preparative reverse-phase high performance liquid chromatography (RP-HPLC). Full characterization of all synthetic AIPs has been reported previously (Gless et al., 2019). The sequences and quality of the synthetic AIPs can be found in Supplementary Table S1.

### Adhesion Assay

This assay was carried out as previously described (Baldry et al., 2016a). Ninety-six well plates were pre-coated with 100 μL/well of 10 μg/mL fibronectin (Fibronectin from human plasma, F2006, Sigma-Aldrich) and incubated for up to 24 h with mild shaking at 4°C. Respective overnight cultures of *S. aureus* strains 8325-4, 61599 (CC398 strain), HG001, HG003 and two *S. aureus* pig isolates (from this study) were diluted 1:100 and grown till OD_600_ = 0.5 in fresh TSB medium, after which the bacteria were treated with the synthesized AIPs belonging to *Staphylococcus hyicus* (10^–4^ mM), *Staphylococcus simulans* (10^–4^ mM), and *Staphylococcus chromogenes* (10^–3^ mM) separately, and grown at 37°C with shaking until OD_600_ = 1.7. The concentrations resulting in 100% inhibitory effect on the *agr* system were chosen according to their IC_50_ values. After removing and washing, untreated and treated *S. aureus* were added to fibronectin-coated wells and incubated statically at 37°C for 1 h. To avoid the toxic effect of DMSO on bacterial growth, the final solvent concentration of DMSO was maintained at 0.2% (v/v) for all experimental and control cultures. After removing the non-adhered bacteria and washing the wells, the attached bacteria were fixed with 2.5% glutaraldehyde in PBS statically for 1 h at 37°C. Binding activity of *S. aureus* was quantified by measuring the OD_5__7__0_ absorbance of resuspension in 96% ethanol after staining with 0.1% crystal violet at room temperature for 30 min. Arbitrary binding units were calculated by dividing the crystal violet absorption OD by the bacterial cell density of 1.7.

### Static Biofilm Assay

As previously described (Nielsen et al., 2012; Goetz et al., 2017) and with minor modifications, overnight cultures were adjusted to OD600 = 0.2 in TSB and then further diluted 1:100 in 66%TSB supplemented with 0.2% glucose. A total of 200 μL of the bacterial suspensions were added to wells where either *S. aureus* 8325-4 WT, 8325-4Δ*agr* (*agr*^–^ strain), CoNS alone, or a 1:1 ratio of *S. aureus* + CoNS was added. After a 24–30 h incubation period, the medium was removed from each well; the plates were washed and allowed to air dry. Dried biofilms were stained with 125 μL of 0.1% crystal violet solution for 30 min, washed three times with PBS and allowed to dry. To quantify the biofilm formation, the stained biofilm was solubilized in 200 μL of 95% ethanol for 10–15 min and 100 μL were transferred to a new microtiter plate, after which the absorbance was measured at 590 nm. In this assay, three biological replicates were performed with eight technical replicates per experiment. Parallel samples were set for CFU quantification by subsequent plating on SaSelect^TM^ plates (Bio-Rad).

### DNA Sequence Analysis

From purified DNA a sequencing library was generated using Nextera XT (Illumina) followed by (2 × 150 bp) paired-end sequencing on a NextSeq (Illumina) instrument. Genome sequences were *de novo* assembled using *skesa* with default settings (Souvorov et al., 2018). From assembled draft genomes the species were identified using the *tuf* gene, which has previously been described to discriminate between *Staphylococcus* species (McMurray et al., 2016; Strube et al., 2018).

### Statistical Analysis

Where applied, we used a 1-way ANOVA analysis (GraphPad Prism version 7.04 software; GraphPad Software Inc., La Jolla, CA, United States). Differences were considered statistically significant at *P* < 0.05.

## Results

### Nasal Colonization of *S. aureus* and Other Staphylococcal Species

To investigate CoNS strains colonizing pig nares we collected nasal swabs from 25 pigs originating from Danish organic pig farms and isolated staphylococcal species on Sa Select^TM^ plates. In total, 384 isolates were obtained of which 75 were identified by MALDI-TOF MS. *tuf* gene analysis and genome sequencing were performed to further verify some of the strains (Hwang et al., 2011; Loonen et al., 2012). Of the 75 isolates 21 were identified as *S. aureus*; corresponding to just over half of the swabs being positive for *S. aureus* (52%; 13/25 pigs). The remaining 54 isolates were identified and classified into 8 CoNS species originating from 20 of the 25 pigs (Table 2). *Staphylococcus sciuri* (40%) was the most dominant amongst the CoNS isolated, followed by *Staphylococcus lentus* (24%), *Staphylococcus xylosus* (24%), *S. simulans* (20%), *S. hyicus* (16%), *Staphylococcus arlettae* (16%), *S. chromogenes* (8%), and finally *Staphylococcus agnetis* (4%). These results show that there is substantial variation among pigs with respect to staphylococcal colonization and that they are commonly colonized by more than one species.

**TABLE 2:** Frequency and presence of eight staphylococcal species isolated from 25 pigs.

**Pig No.**	**% Isolation frequency – *species identity* – species isolated/pig**								
	
	**40%**	**24%**	**24%**	**16%**	**16%**	**20%**	**8%**	**4%**	**52%**
	***S. sciuri***	***S. lentus***	***S. xylosus***	***S. hyicus***	***S. arlettae***	***S. simulans***	***S. chromogenes***	***S. agnetis***	***S. aureus***
1	X		X						X
2				X		X			X
3	X					X			X
4	X			X		X			X
5				X		X			X
6	X								X
7			X						X
8							X		X
9									X
10									X
11									X
12									X
13									X
14		X			X				
15	X					X			
16		X							
17		X	X						
18	X	X			X				
19		X			X				
20	X	X							
21				X			X	X	
22	X				X				
23	X		X						
24	X		X						
25			X						

*Rows indicate the presence of staphylococci in the individual pigs. Columns contain the different staphylococcal species identified.*

### *S. aureus* Virulence Factor Expression Is Affected by CoNS Strains

**FIGURE 1:**
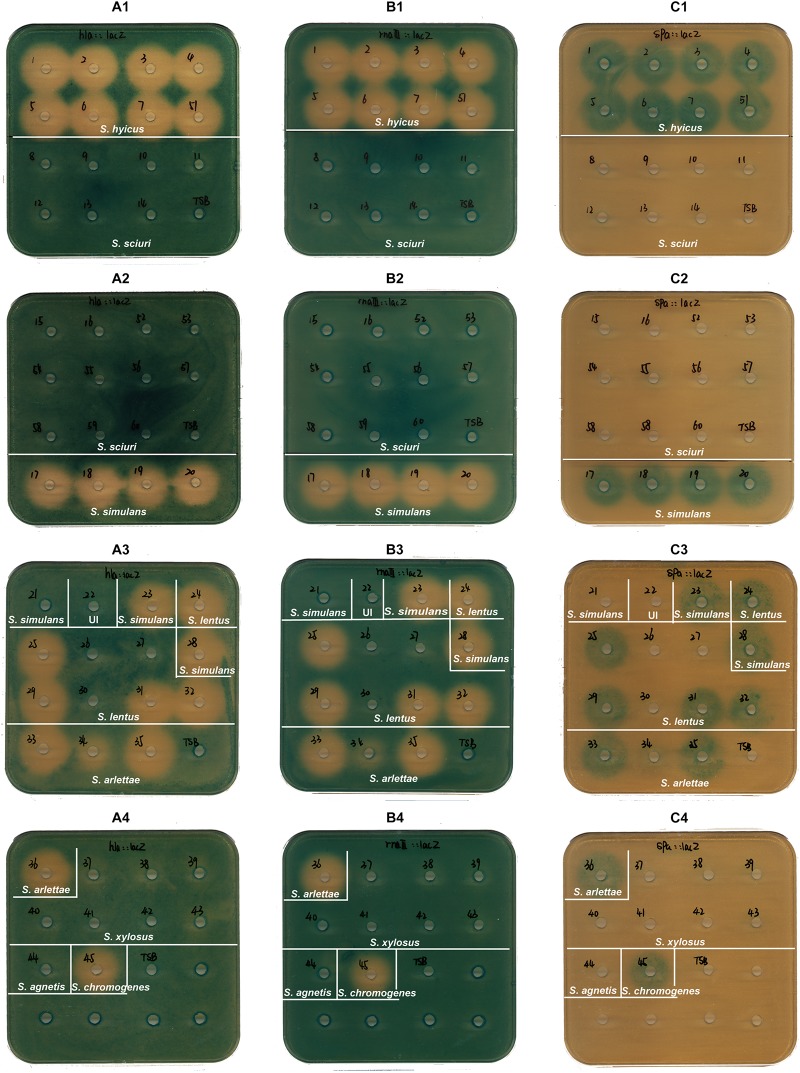
Effect on virulence factor expression of *S. aureus* by CoNS culture supernatant. TSA agar plates (with erythromycin and X-gal) containing the *hla*:lacZ (PC322; Ery^r^) (plates **A1–A4**), the *rnaIII*:*lacZ* (SH101F7; Ery^r^) (plates **B1–B4**), or the *spa*:*lacZ* (PC203; Ery^r^) (plates **C1–C4**) reporter strains of *S. aureus* were used to screen the cell-free overnight culture supernatants from 55 isolates. Sixty microliter of supernatant or TSB (as a negative control) were added to the wells in the plates. The supernatants in wells are from *S. hyicus* (wells 1–7 and 51); *S. sciuri* (wells 8–16 and 52–60); *S. simulans* (wells 17–21, 23 and 28); *S. lentus* (wells 24–27 and 29–32); *S. arlettae* (wells 33–36); *S. xylosus* (wells 37–43); *S. agnetis* (well 44); *S. chromogenes* (well 45); UI shown in well 22 stands for an unidentified isolate. The plates were incubated at 37°C for 10–24 h (until zones appeared). The assay was performed three times as biological replicates. This figure is representative of one set of screening plates.

Based on previous reports of cross-communication between *S. aureus* and CoNS strains we hypothesized that *S. aureus* interacts via *agr* with the surrounding microbial consortia including the resident CoNS. Therefore, the secreted products of the isolated CoNS strains were screened for their ability to modulate *S. aureus agr* using a previously established reporter assay. This assay is based on three reporter strains where the promoters of RNAIII, *hla* and *spa*, respectively, are fused to *lacZ* (Nielsen et al., 2010). Upon induction of *agr* such as is observed during entry into stationary growth phase, promoters of both RNAIII and *hla* will be induced while that of *spa* will be repressed. Therefore, after incorporation of the reporter strains together with the LacZ substrate into the agar plates they will become blue when containing the RNAIII and *hla* reporter strain fusions, but will remain colorless when containing the *spa* reporter strain after overnight incubation. Conversely, if an *agr* inhibiting compound has been added to a well in the agar plate reduced expression of *hla* and RNAIII but increased expression of *spa* will be observed. As seen in Figure 1, the extent to which CoNS supernatants affected the *S. aureus agr* (*agr*-I) system varied between species and in some cases even within species. Interestingly, while *S. sciuri* was the most prevalent species in the swabs, none of the supernatants from isolates of this species affected the *S. aureus agr* system. In contrast, isolates belonging to *S. hyicus*, *S. simulans*, and *S. lentus* species contained the most isolates with *S. aureus agr* modulation capabilities. These findings show that CoNS display varying ability to repress the *S. aureus agr* and that such repression is commonly observed.

### Effect of CoNS Strains on *S. aureus agr* Groups I–IV

**FIGURE 2:**
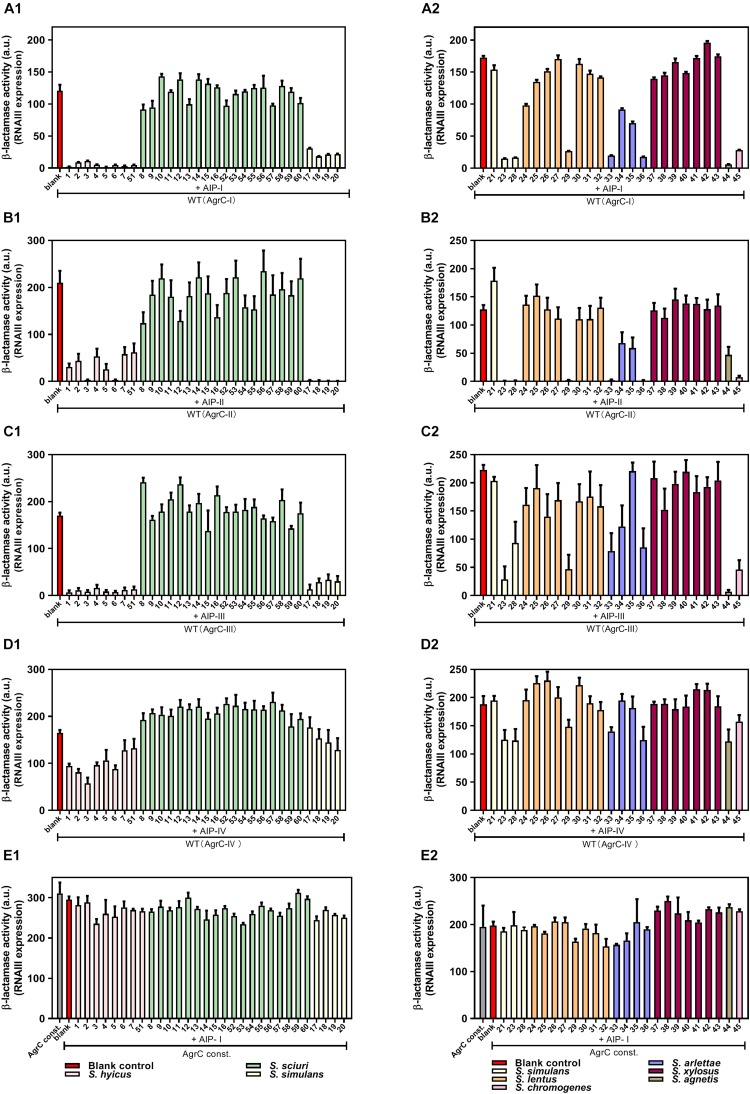
AgrC-mediated interference of RNAIII expression by CoNS supernatants. Reporter strains RN10829 (P2-*agrA*: P3-*blaZ*)/p*agrC*-I-IV (WT) **(A1–D2)** and RN10829 (P2-*agrA*: P3-*blaZ*)/p*agrC*-I-R23H (AgrC const.) **(E1,E2)** were grown to OD600 = 0.35, and exposed to 1/10 volume supernatant of CoNS and 1/10 external inducing AIP-I-IV supernatant. After 45 min incubation, RNAIII expression was assessed using the nitrocefin hydrolysis method and analyzed for relative β-lactamase activity by nitrocefin conversion. The numbers displayed on the X-axis correspond to those in Figure 1: No. 1–7 and 51 (*S. hyicus*); No. 8–16 and 52–60 (*S. sciuri*); No. 17–21, 23 and 28 (*S. simulans*); No. 24–27 and 29–32 (*S. lentus*); No. 33–36 (*S. arlettae*); No. 37–43 (*S. xylosus*); No. 44 (*S. agnetis*); No. 45 (*S. chromogenes*). Each CoNS species is represented by a different color. Each column is representative of at least three biological replicates and the error bars represent the standard deviation.

In the agar plate assay (Figure 1) we had determined the inhibitory activity of CoNS strains in a *S. aurues* strain belonging to AgrC group I. To determine if the CoNS AIPs are able to inhibit *agr* in *S. aureus* strains carrying the AgrC groups II to IV, and to obtain a quantative measure of the inhibitory effect we employed β-lactamase reporter strains that monitor expression of the RNAIII P3 promoter in cells expressing AgrC groups I to IV. As these strains have been engineered so that they do not produce intrinsic AIPs, induction of *agr* requires addition of supernatants from strains producing the corresponding AIP group (Nielsen et al., 2014). In this system, the activity of the reporter strains were measured in the presence or absence of cell-free, overnight culture supernatants of our CoNS isolates. Importantly, all the CoNS supernatants that displayed an *agr*-inhibitory activity in the plate assay also inhibited RNAIII expression in *S. aureus* strains carrying *agr* groups I, II, and III, whereas the inhibitory potential against group IV was only marginal (Figures 2A1–D2). Further we confirmed the notion that the CoNS AIPs affect *S. aureus agr* via competitive inhibition of AgrC as we saw little to no inhibition of the P3 promoter in a reporter strain encoding a constitutively active AgrC variant of *agr* group I that displays kinase activity in the absence of inducing AIP (Geisinger et al., 2009) (Figures 2E1,E2).

In addition to inhibition, we were also interested in exploring whether any of the staphylococcal supernatants could induce *S. aureus agr* activity. To this end we tested the ability of our CoNS isolates to induce *S. aureus agr* using the same β-lactamase reporters of the *S. aureus agr* groups, but in this case the staphylococcal supernatants were used as presumptive inducers omitting induction by the cognate AIP. Our results show that none of the CoNS supernatants were capable of activating any of the four *S. aureus agr* groups (Supplementary Figure S1). These results show that CoNS strains interfere with *agr* induction by competing with the *S. aureus* AIPs for AgrC binding and that they generally have inhibitory activity toward *S. aureus agr*.

### Dual Species Biofilm Involving *S. aureus* and CoNS

**FIGURE 3:**
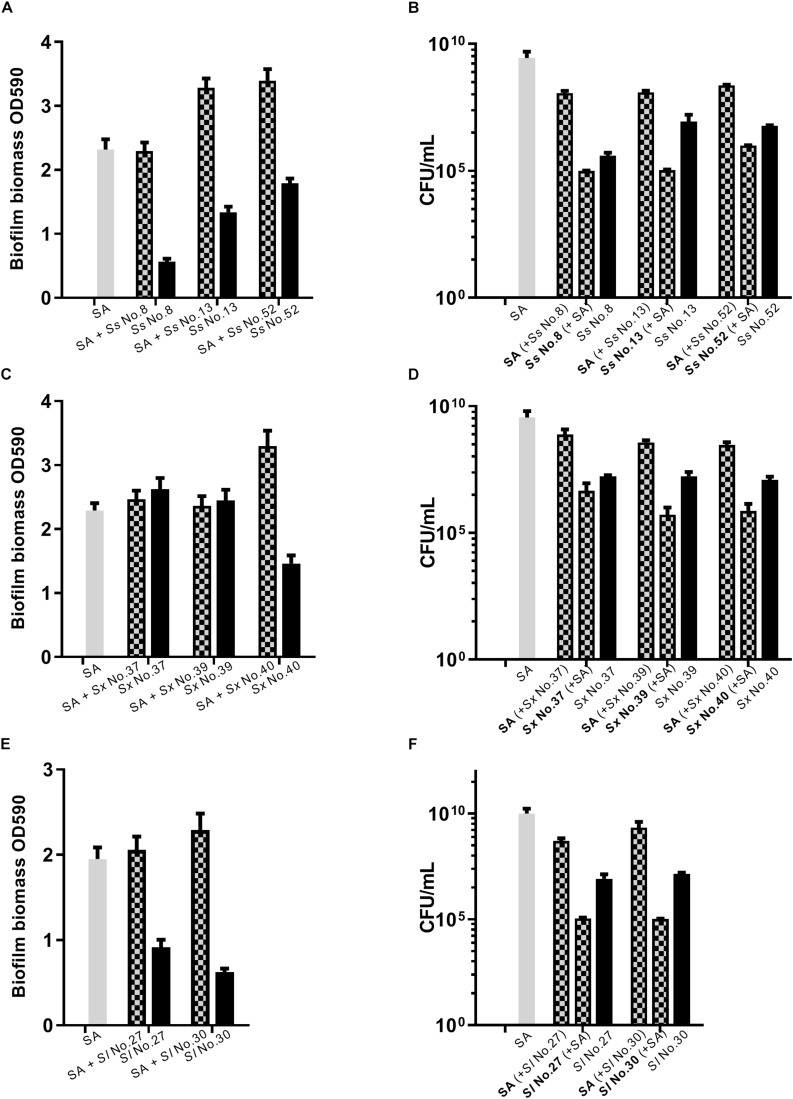
Dual species biofilm formation between *S. aureus* and CoNS strains not displaying *agr* inhibitory activity. For dual-species biofilms, *S. aureus* 8325-4 (SA) was co-cultured together with one of *S. sciuri* (*Ss*, **A,B**), *S. xylosus* (*Sx*, **C,D**) and *S. lentus* (*Sl*, **E,F**) and biofilm biomass **(A,C,E)** or CFU **(B,D,F)** were determined as indicated by mix color bars and compared to biofilms formed by the individual species (SA indicated by gray bars and CoNS by black bars).

**FIGURE 4:**
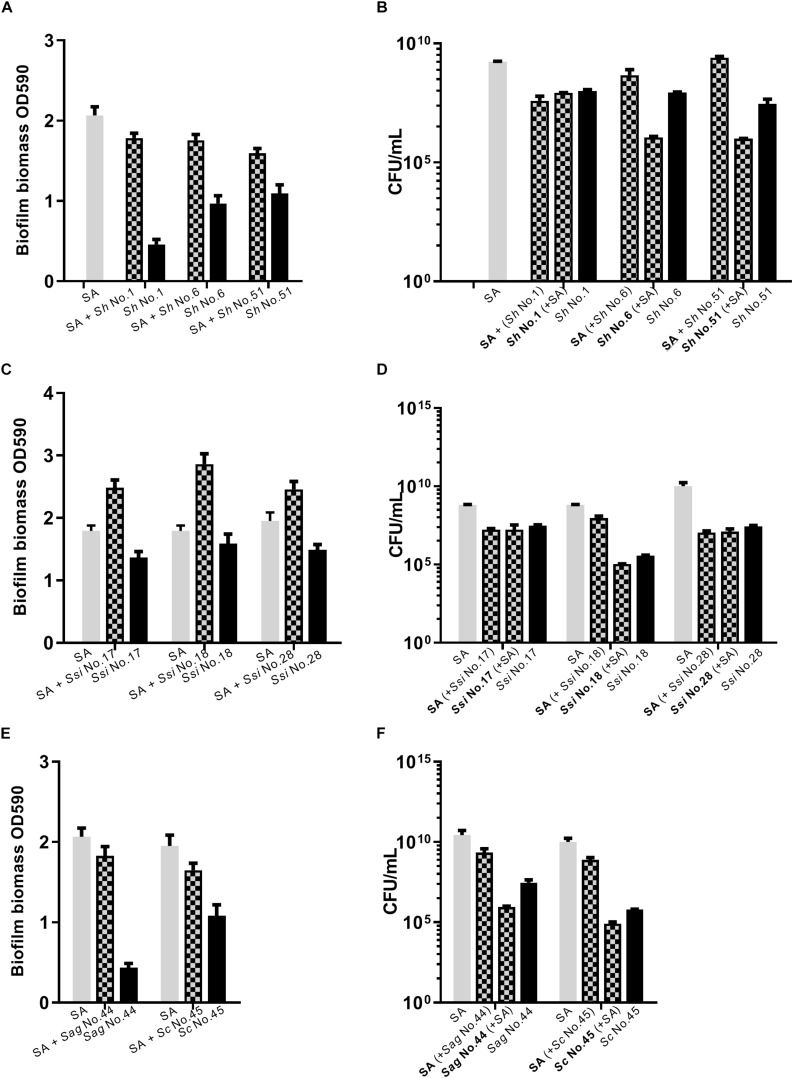
Dual species biofilm formation between *S. aureus* and CoNS strains displaying *agr* cross-inhibition. For dual-species biofilms, *S. aureus* 8325-4 (SA) was co-cultured together with one of *S. hyicus* (*Sh*, **A,B**), *S. simulans* (*Ssi*, **C,D**), *S. agnetis* and *S. chromogenes* (*Sag*/*Sc*, **E,F**) and biofilm biomass **(A,C,E)** or CFU **(B,D,F)** were determined as indicated by mix color bars and compared to biofilms formed by the individual species (SA indicated by gray bars and CoNS by black bars).

**FIGURE 5:**
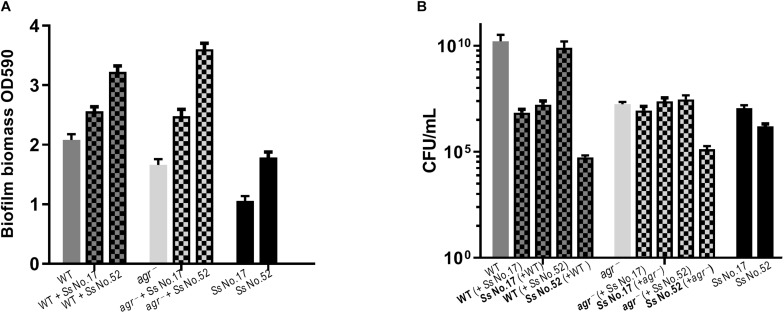
Biofilm formation does not correlate with *agr* inhibition. For dual-species biofilms, *S. aureus* 8325-4 WT or *agr*^–^ (Δ*agr*) mutant strains were co-cultured together with either *S. simulans* (Ss No. 17) or *S. sciuri* (Ss No. 52) and biofilm biomass **(A)** or CFU **(B)** were determined as indicated by mix color bars and compared to biofilms formed by the individual species (SA indicated by gray bars and CoNS by black bars).

As *S. aureus agr* is known to influence biofilm formation (Le and Otto, 2015), we asked if CoNS strains potentially producing *agr* repressing peptides affected biofilm formation. When grown individually, the CoNS strains were less robust at forming biofilm than *S. aureus* (Figures 3, 4 and Supplementary Figure S2). From these we selected 1-3 CoNS strains from each species to examine biofilm formation in the presence of *S. aureus.* While the biofilm biomass was quantified by crystal violet staining, bacterial composition of these dual-species biofilms was determined by inspection of CFU on SaSelect^TM^ plates. In all, we tested biofilm formation for eight combinations where the CoNS species had no inhibitory effect on *S. aureus agr* (Figure 3), eight combinations for those CoNS species with a strong *S. aureus agr* inhibitory effect (Figure 4), and another three combinations with CoNS strains with varying *agr* inhibitory effects (Supplementary Figure S2). When examining the composition of dual-species biofilm (Figures 3B,D, 4B,D and Supplementary Figure S2B), both species were represented. For 8 out of the 19 dual-species combinations we observed increased biofilm biomass when compared to biofilm formation by individual strains. Importantly, these grouped almost evenly into the *agr* cross-inhibition group (5 out of 10) and the non-inhibitory group (3 out of 9). While this data already indicated that the increased biofilm in dual species biofilms was independent of *agr* cross-talk, we sought to consolidate this finding further. For this we chose one strain capable of *agr* cross-inhibition (*S. simulans* No. 17) and one strain from the non-inhibitory group (*S. sciuri* No. 52), and analyzed their effect on biofilm formation of a *S. aureus agr* mutant strain. These data indicate that the absence of a functional *agr* in *S. aureus* did not influence biofilm formation when mixed with CoNs strains (Figure 5). Collectively our data show that the presence of both *S. aureus* and CoNS may in some instances enhance biofilm formation when compared to that formed by the individual strains, but in those cases it is unrelated to *agr* mediated interactions.

### Strain-Specific Enhancement of *S. aureus* Adherence to Fibronectin in the Presence of Synthesized CoNS AIPs

**FIGURE 6:**
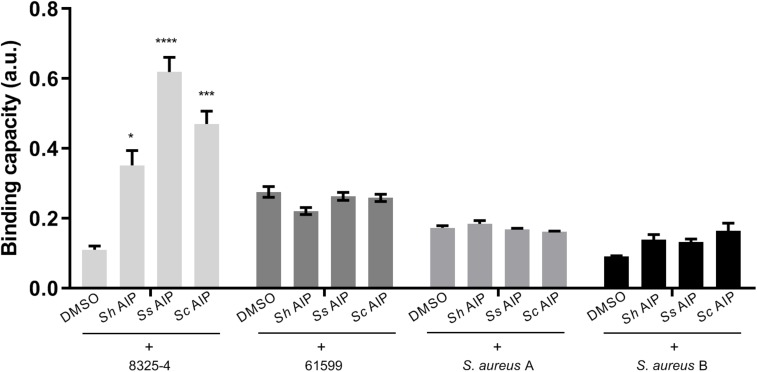
Synthesized AIPs from *S. hyicus* (*Sh* AIP), *S. simulans* (*Ss* AIP), and *S. chromogenes* (*Sc* AIP) enhance *S. aureus* adherence to host factors fibronectin *in vitro*. *S. aureus* strains were separately treated with AIPs (10^–4^ mM *S. hyicus*, 10^–4^ mM *S. simulans* AIPs and 10^–3^ mM *S. chromogenes*), incubated and fixed in human fibronectin pre-coated 96-well plates. Crystal violet staining was performed to quantify the amount of *S. aureus* adhering to host factors via measurement of OD_590_. Results shown are representative of 3 independent experiments. Each bar represents the average of 8 biological replicates and the error bars represent the standard deviation. ^∗^*P* < 0.05; ^∗∗∗^*P* < 0.001; ^****^P < 0.0001.

As inhibition of *S. aureus agr* leads to increased expression of surface adhesion proteins recognizing host factors, we were curious to see whether the addition of synthesized AIPs from CoNS would lead to increased binding of *S. aureus* to host factor. To address this, we studied the fibronectin binding capacity of different *S. aureus* strains namely the laboratory strain 8325-4 (CC8), the livestock associated CC398 strain 61599 (Tang et al., 2017b) as well as two *S. aureus* strains identified from the pig nares together with either *S. chromogenes* (A) or *S. hyicus* and *S. simulans* (B). *S. aureus* A was classified as CC8 and *S. aureus* B as CC45. Strains belonging to both CC8 and CC45 have previously been found associated with live stock (Tang et al., 2017a). When these strains were separately treated with synthesized AIPs of *S. hyicus*, *S. simulans*, and *S. chromogenes*, that have been detected in a previous study (Gless et al., 2019), we observed a significant increase in *S. aureus* 8325-4 binding to fibronectin in the presence of the CoNS AIPs, in comparison to the vehicle (DMSO)-treated control (Figure 6). However, neither strain 61599 nor the pig-derived *S. aureus* isolates obtained in this study and tested here were affected by the presence of CoNS AIPs over the vehicle control. In consideration of the known regulatory defects of *S. aureus* 8325-4, we also examined the adherence of repaired strains HG001 (restored *rsbU*, an activator of SigB) and HG003 (restored *rsbU* and *tcaR*, an activator of protein A transcription) under the same condition (Herbert et al., 2010) (Supplementary Figure S3). Both of these strains showed a low adhesion to fibronectin. Thus, exposure to CoNS AIPs does not lead to increased binding to fibronectin except for strain 8325-4.

## Discussion

Coagulase negative staphylococci comprise a diverse group of staphylococcal species that largely are harmless colonizers of both humans and animals. For a given host several CoNS are commonly present and the composition varies both within and between host species (Nagase et al., 2002). Likewise, pigs have been reported to be colonized by a variety of CoNS with one study describing 10 species including *S. hyicus*, *Staphylococcus haemolyticus*, *Staphylococcus warneri*, *S. simulans*, *S. xylosus*, and *S. sciuri* to be isolated at approximately equal frequency (Nagase et al., 2002). Others document higher CoNS species numbers (between 18 and 20 different CoNS) including the afore-mentioned, as well as *S. saprophyticus* and *S. cohnii* (Schoenfelder et al., 2017; Verstappen et al., 2017). However, both the latter studies report a marked increase in *S. sciuri* prevalence over the other species amounting to between 30 and 46% of the total colonizing CoNS species (Schoenfelder et al., 2017; Verstappen et al., 2017). In our investigation we also found *S. sciuri* to be the most prevalent CoNS being isolated from 40% of the pigs followed by *S. lentus* and *S. xylosus.* Unlike the Verstappen study though, we did not isolate *S. saprophyticus* or *S. cohnii* which were the other most prominent species identified along with *S. sciuri* (Verstappen et al., 2017).

Interestingly CoNS strains appear to be common producers of AIP molecules that resemble the AIPs of the *S. aureus agr* quorum sensing system. Analogous to the cross-inhibition of *agr* that occurs between *S. aureus* strains belonging to different AgrC subgroups, the CoNS AIPs also tend to inhibit expression of *agr* controlled genes. Previously we showed that out of 52 staphylococcal isolates obtained from a common strain collection, 37 were capable of inhibiting *agr* of *S. aureus* representing 17 different CoNS species (Canovas et al., 2016). Here, we aimed to investigate the extent to which CoNS strains isolated form the same niche environment (i.e., from individual pigs) were able to repress *S. aureus agr*. Our results show that out of 25 pigs we isolated 8 different CoNS species of which 24 out of 54 strains had quorum quenching properties. Interestingly out of 18 tested *S. sciuri* strains none were able to repress *agr*. Similar was observed for *S. xylosus* whereas for *S. lentus*, which was present in 24% of the pigs, *agr* was repressed by some strains but unaffected by others. In contrast, all isolates of both *S. hyicus* and *S. simulans* displayed strong *agr* repressing activity. Using a constitutively active AgrC variant we were able to show that the CoNS strains likely repress *agr* through production of AIP-like molecules that are secreted to the culture supernatant and compete with *S. aureus* AIPs for binding to *S. aureus* AgrC, as opposed to other *agr* quorum quenching mechanisms such as via AgrA binding (Baldry et al., 2016b). This notion was confirmed by synthesis of selective CoNS AIP molecules. Furthermore, we observed correlation between the inhibitory potential of individual CoNS strains against *S. aureus agr* group I and the inhibition exerted on groups II and III, while the inhibition pattern was not clearly reflected on group IV. Low inhibitory activity against *agr* group IV for entities that are highly active against other groups have been described before (Gless et al., 2019). We also examined if any of the CoNS strains were able to induce the *S. aureus agr* system; however, none of the strains demonstrated such activity. For *P. aeruginosa*, analogs of quorum sensing molecules have been reported to induce quorum sensing (Smith et al., 2003) and we have observed that synthesized AIPs of *S. schleiferi* and *Staphylococcus hominis* are capable of inducing *S. aureus* AgrC group IV (Gless et al., 2019). However, cross-species induction of *agr* appears to be a rare phenomenon and the vast majority of *agr* modulating compounds interfere with quorum sensing induction (Hansen et al., 2018).

Previously it has been suggested that presence or absence of CoNS strains may correlate with *S. aureus* colonization. For example, Verstappen et al. observed a lower frequency of *S. aureus* colonization in the presence of *S. sciuri*, *S. cohnii*, or *S. saprophyticus*. We did not identify any *S. cohnii* or *S. saprophyticus* in our sampled pigs, and out of the 10 pigs positive for *S. sciuri*, 4 were co-isolated with *S. aureus* while 6 were not. However, we did observe that all *S. arlettae* and *S. lentus* isolates were colonizing pig nares where *S. aureus* was not found to be present. To investigate competitive behavior between CoNS strains and *S. aureus* we performed a series of dual-species biofilm studies based on the notion that *agr* has been reported to influence both biofilm formation and dispersal (Boles and Horswill, 2008; Periasamy et al., 2012). This rational was also made interesting by the recent observations by Gonzalez et al. that *S. epidermidis* secreted soluble products (when added to *S. aureus* cultures) inhibit *S. aureus* biofilm formation, but when the two species are co-inoculated and grow in physical contact they are capable of forming a robust dual-species biofilm (Gonzalez et al., 2018). Our data corroborate Gonzalez’s observations in that we also show robust biofilm formation in the dual-species setting with no evident out-competition of one over the other species. Such dual-species interactions can benefit both species in that they can persist in a colonizing state more robustly, as biofilms are extremely hard to eradicate by both the host and by antimicrobial therapies, and thus also providing a constant reservoir for possible *S. aureus* chronic infections (Archer et al., 2011). Moreover, even though we have observed the effect of interaction between *S. aureus* and CoNS on biofilm formation, no correlation to *agr*-inhibition was seen. Further studies are needed to better understand the complexity of these interactions.

In context of antibacterial therapy, cross-talk between staphylococci and *S. aureus* via *agr* has become a topic of interest. We recently showed that *agr* inhibition by AIP-like molecules reduces *S. aureus* induced lesions in an atopic dermatitis model (Baldry et al., 2018) and this was supported by the finding that CoNS strains reduce skin barrier damage by inhibiting production of proteases and phenol-soluble modulins secreted by *S. aureus* (Williams et al., 2019). Another study reports a synthetic AIP from *Staphylococcus caprae* that dramatically reduced dermonecrotic injury caused by *S. aureus* and reduced cutaneous bacterial burden relative to controls (Paharik et al., 2017). However, as inhibition of *agr* is associated with increased expression of surface adhesion proteins favoring host adhesion and immune evasion (Novick et al., 1993), one could speculate that CoNS strains may increase the ability of *S. aureus* to colonize. For only one strain of *S. aureus*, namely 8325-4, we observed a significant increase in adhesion to human fibroncetin in the presence of CoNS AIPs. This was not seen for the livestock associated MRSA strain 61599 belonging to CC398, the two *S. aureus* isolates obtained from pigs in the present study, nor for the strains HG001 or HG003 that are derivatives of 8325-4 and restored by *rsbU* or *rsbU*/*tcaR* regulatory genes. Thus, we could not consistently demonstrate an effect of CoNS AIPs on *S. aureus* binding to fibronectin. In conclusion, the interactions between coagulase-negative staphylococci and *S. aureus* are complex and involve both *agr* dependent and independent factors, which future studies will be required to elucidate.

## Conclusion

We have conducted an investigation of the possible role of the *agr* cross-talk between *S. aureus* and CoNS strains isolated from the same colonizing location. We show that there are substantial variations with respect to species colonization amongst the pig hosts tested, as well as in *S. aureus agr*-modulation capacity of isolated CoNS species. Importantly, our results document multiple interactions between *S. aureus* and CoNS and they suggest that *S. aureus* adhesion and dual-species biofilm formation can indeed be influenced by CoNS in both *agr*-dependent and *agr*-independent manners.

## Data Availability Statement

All datasets analyzed for this study are included in the manuscript/Supplementary Files.

## Ethics Statement

No permission is required to sample the nostril of pigs according to the Danish Animal Experimentation Act § 1.2.

## Author Contributions

PP, MB, MSB, and HI designed the study. PP, BG, SB, and CE-G conducted the experimental work. PP, MB, MSB, HI, CO, BG, PA, CE-G, and SB analyzed the data. PP, MB, MSB, BG, CO, and HI wrote the manuscript.

## Conflict of Interest

The authors declare that the research was conducted in the absence of any commercial or financial relationships that could be construed as a potential conflict of interest.
