# Supplementary material for: Effect of Co-inhabiting Coagulase Negative Staphylococci on S. aureus agr Quorum Sensing, Host Factor Binding, and Biofilm Formation
Source: Front Microbiol. 2019 Sep 27;10:2212. doi: 10.3389/fmicb.2019.02212 (PMC6777374; doi:10.3389/fmicb.2019.02212)
Supplement: Supplementary file 1 [file Table_1.DOCX]

Supplementary Material

Effect of co-inhabiting coagulase negative staphylococci on *S. aureus agr* quorum sensing, host factor binding, and biofilm formation

Pai Peng^1^, Mara Baldry^1^, Bengt H. Gless^2^, Martin S. Bojer^1^, Carmen E. Gongora^1^, Sharmin J. Baig^3^ , Paal S. Andersen^1,3^ , Christian A. Olsen^2^, Hanne Ingmer^1*^

*** Correspondence:** Hanne Ingmer: [hi@sund.ku.dk](mailto:hi@sund.ku.dk)

# Supplementary Data

**Identification of CoNS by DNA sequencing analysis.**

The majority of the CoNS isolates in this study were successfully identified by MALDI-TOF MS, but for the few unmatched isolates *tuf* gene and genome sequencing were performed for correct identification (the sequencing data is not shown). No.23 and No.28 were identified as *S. simulans*. No *tuf* gene could be found in strain No.22, so it has been described as unidentified (UI).

# Supplementary Figures

**
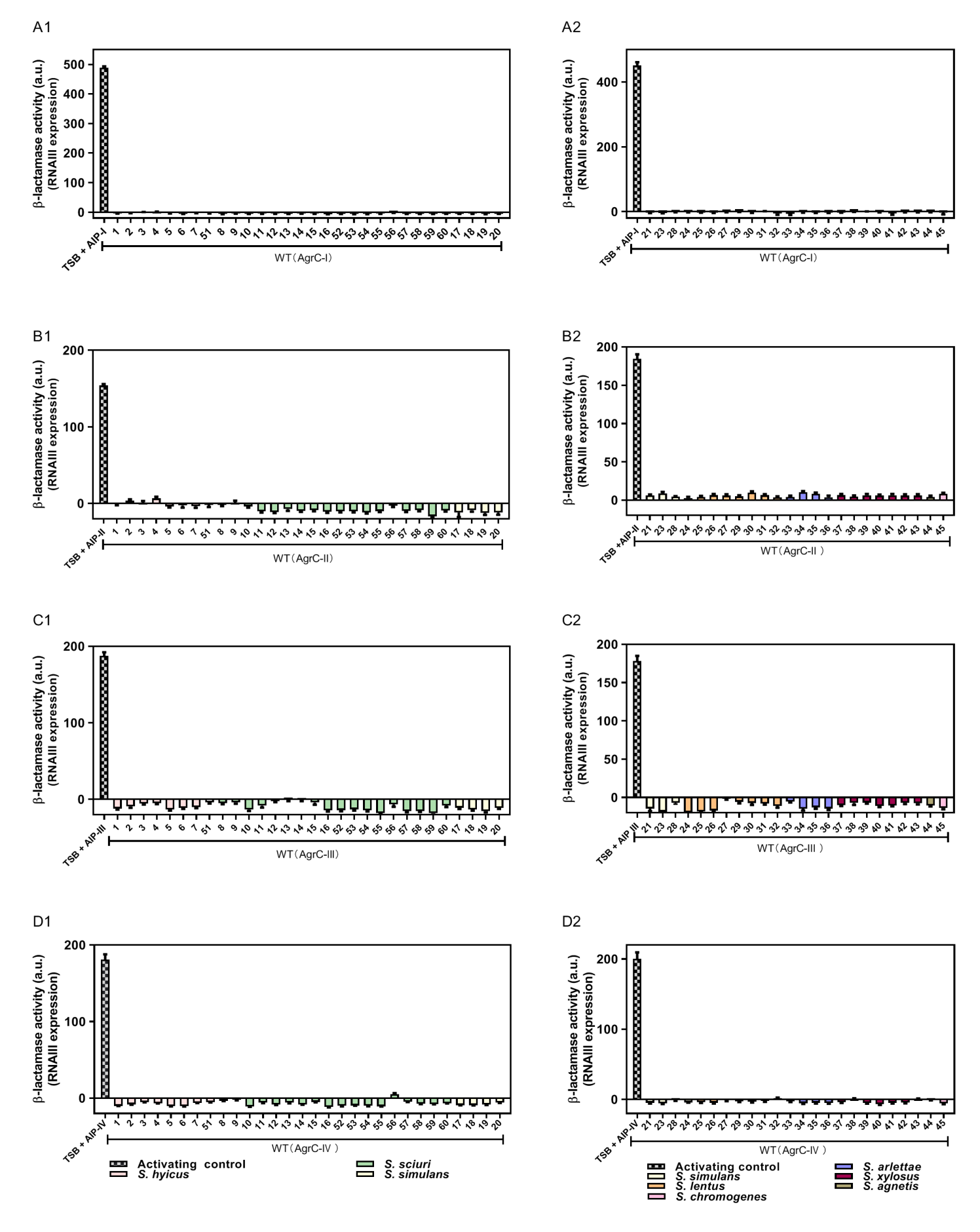
**

**Supplementary Figure S1.** *agr* is not activated by CoNS supernatants from the four *S. aureus agr* groups as monitored by RNAIII expression. Reporter strains (P2-*agrA*: P3-*blaZ*)/p*agrC*-I-IV **(A1-D2)** were exposed to 1/10 volume supernatant of CoNS as the external AIP, TSB+AIP-I-IV as activating controls. Relative β-lactamase activity of these reporter strains were analyzed for RNAIII expression of *S. aureus* by nitrocefin conversion. The numbers displayed on the X-axis correspond to those in Figure 1: No.1-7 and 51(*S. hyicus*); No.8-16 and 52-60 (*S. sciuri*); No.17-21, 23 and 28 (*S. simulans*); No.24-27 and 29-32 (*S. lentus*); No.33-36 (*S. arlettae*); No.37-43 (*S. xylosus*); No.44 (*S. agnetis*); No.45 (*S. chromogenes*). Each species of CoNS is represented by different colours. Each column is representative of at least three biological replicates and the error bars represent the standard deviation.

**
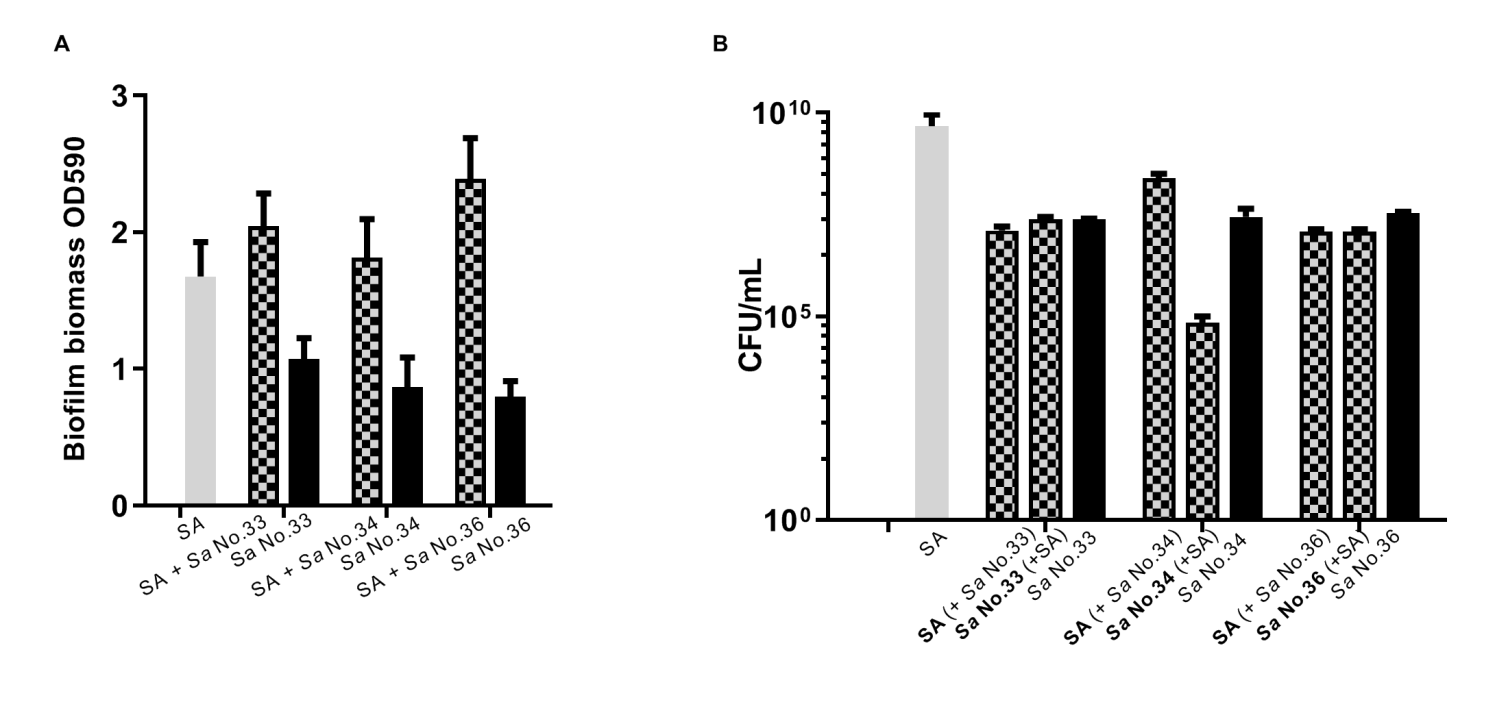
Supplementary Figure S2** Interaction in dual-species biofilms between *S. aureus* (SA) and CoNS species *S. arlettae* displaying differential inhibitory effect on *agr* system. For dual-species biofilms, *S. aureus* 8325-4 (SA) was co-cultured together with *S. arlettae* (*Sa*, A and B). Biofilm biomass (A) or CFU (B) were determined as indicated by mix color bars and compared to biofilms formed by the individual species (SA indicated by grey bars and CoNS by black bars).


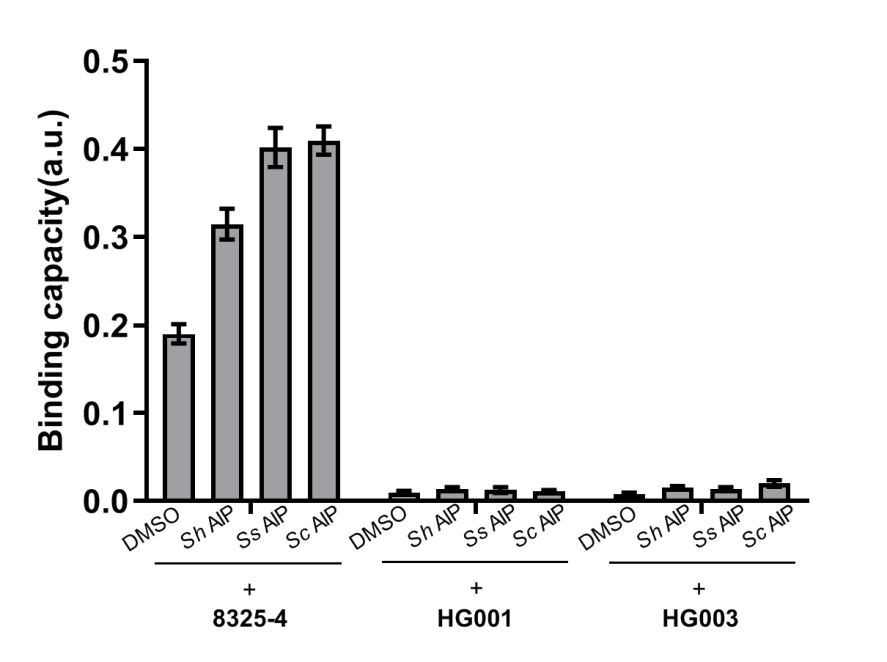


**Supplementary Figure S3** Restored strains of 8325-4, HG001 (restored *rsbU*) and HG003 (restored *rsbU* and *tcaR*) exposed to CoNS synthesized AIPs show low adhesion to host factor fibronectin in vitro. *S. aureus* treated with AIPs (10^-4^ mM *S. hyicus*, 10^-4^ mM *S. simulans* AIPs and 10^-3^ mM *S. chromogenes*) was incubated and fixed in human fibronectin pre-coated 96-well plates. Crystal violet staining was performed to quantify the amount of *S. aureus* adhering to host factors via measurement of OD_590_. The results are representative of 3 independent experiments. Each bar represents the average of 8 biological replicates and the error bars represent the standard deviation.

# Supplementary Tables

**Supplementary Table 1.** The sequences and quality of synthesized AIPs used in the adhesion assay.

| **Staphyloccocus species**  **sequence** | **Purity** | **HPLC traces** | **HRMS *m/z*: [M+H]^+^ Calcd/found** |
| --- | --- | --- | --- |
| *S. hyicus* AIP  (KINP-[CTVFF]) | 95% | λ= 210 nm | C_51_H_76_N_11_O_11_S^+^  1050.5441/ 1050.5411 |
| *S. simulans* AIP  (KYNP-[CLGFL]) | 99% | λ = 210 nm | C_50_H_74_N_11_O_11_S^+^  1036.5284/ 1036.5257 |
| *S. chromogenes* AIP (SINP-[CTGFF]) | 95% | λ= 210 nm | C_45_H_63_N_10_O_12_S^+^  967.4342/ 967.4337 |
